# Institutional Notes and News

**Published:** 1910-02-26

**Authors:** 


### Institutional Notes and News.

**The King and Liverpool.**

His Majesty the King has graciously signified his pleasure that the Liverpool Country Hospital for Children be known for the future as Royal. This Hospital has only been in possession of its new buildings at Heswall for about a year, although it has carried on work in temporary quarters for ten years previously. The Committee is naturally very gratified at the honour which has been conferred on the hospital, and at this recognition of the work which it has carried on. This was the pioneer institution of its kind in the country, and the Committee is extremely pleased that the name of Liverpool has been identified with the movement.

**Royal Bucks Hospital: Changes.**

At the recent annual general meeting of the Royal Buckinghamshire Hospital, the chairman, Mr. George Carrington, had to explain how an overdraft of 13s. 8d. had increased in the course of last year to one of £157, while the receipts for the same period had amounted to £110 more than usual. The new wing, it appears, which was visited and praised by Sir Frederick Treves last July, is responsible for the bulk of this overdraft, as the coal bill has increased, a staff nurse has been appointed, *x*-ray treatment is now given, and first-class operations are now performed which add to the expense in every way. Unfortunately, moreover, the debt on the new wing itself is £2,120; but against this must be set a promise of £1,000 on condition that the balance is provided. Passing from finance to patients, the report stated that 411 in-patients were admitted (surgical cases showing a preponderance of rather more than two to one), and 1,227 out-patients; each class had increased roughly by 100 on the number admitted in 1908. The Carrington Convalescent Fund has done good work by finding the expenses of convalescent treatment at different homes for ten patients. The two legacies which had been received during the year amounting to over £600, have been invested in Consols. Mr. Carrington further pointed out that not only was a clean balance-sheet wanted, but also a new servants' hall and an improved larder. "The last £1,000," he said, "is always the hardest to raise."

**Aberdeen Royal Infirmary.**

The process of applying legacies to meet current expenses, or rather to reduce current debt, looks as if it were to become an annual feature at the Aberdeen Royal Infirmary. The deficit this latest annual report has brought to public light is one of £1,313, apart from £123 debited to extraordinary expenditure. By the diversion of legacies from the invested funds, which every financially sound public institution likes to increase each year by their means, the deficit was brought down, on paper, to the less deplorable but most unsatisfactory sum of £489, £947 having been received under this head. This loss to the capital account, therefore, of nearly £1,000 is doubly serious. In view of this position, that part of the annual report which deals with work done and money received has peculiar importance. In round figures, 3,000 in-patients were admitted during the year and 16,796 out-patients, while the daily average number of occupied beds was 226. The ordinary income, which was £85 less than in 1908, was £11,635, and the ordinary expenditure that plus, of course, the deficit of £1,313 mentioned already. It is worth noticing, perhaps, that even if the legacies had been what they were in 1908 (£1,410) the institution would still be in debt. In other words, when every scrap of money is used to meet running expenses, these expenses outstrip total receipts. No mention of any reserve fund has reached us, and it does not seem that beyond the habitual pious hope that the public will give "the most liberal support" to the institution any steps have been decided on to meet what is virtually an impossible state of affairs. It may not be out of place, therefore, to remind the friends of Aberdeen Royal Infirmary of an authoritative *obiter dictum* quoted in The Hospital last week that "it is impossible to have a good income without good advertising." Somebody is not doing his business when debt threatens to become a hardy perennial like this.

**Changing a Hospital's Title.**

Sir Richard B. Martin, Bart., Treasurer, who presided at the annual general meeting of the Governors of St. Mark's Hospital for Fistula on Thursday, February 10, in moving the adoption of the report, called attention to the fact that in spite of all efforts to raise funds, and the severe economy which had been practised in all directions, the income for the past twelve months had been £900 less than the expenditure for the same period. In consequence it had been necessary to part with £1,000 out of capital to meet the deficit. This was a very serious matter, considering the very limited amount of invested property held by the trustees. The Chairman drew attention to an important proposal submitted by the Committee of Management, namely, that in future the word "Cancer" should be added to the name of the Hospital, making it read, "The St. Mark's Hospital for Cancer, Fistula, and Other Diseases of the Rectum." This was unanimously agreed to. It was pointed out that the new title would far more fittingly than the old name describe the scope of the hospital's work. A very large proportion of the patients suffered from this malignant disease. The treatment of cancer of the rectum demanded much time and care on the part of both the doctors and the nurses, and this time and care had been very efficiently and effectively rendered by the staff, who were able to bring their special training and knowledge to bear upon the treatment of this distressing malady. It was reported that whereas during 1908 the number of in-patients was 522, in 1909 a total of 627 were admitted, while the number of out-patients in 1909 was 1,812, as compared with 1,725 in 1908, a considerable increase in both departments.

**Dewsbury and District Infirmary.**

To be able to sum up the year's work as Mr. E. D. Booth, the Treasurer of the Dewsbury and District Infirmary did in seconding the report the other day by stating that "we have increased our income and decreased our expenditure," is capital. But this does not mean that the income was sufficient. The figures he gave were an income of £2,826, and an outlay of £2,892, the total deficiency being one of £308. He admitted, however, that the reserve of the children's ward fund had been drawn upon to give them a clear balance sheet. It is hoped that, if the various denominations will make greater efforts than they have hitherto done to furnish worthy contributions, the above process will not be necessary in the future, and the gap between income and expenditure ultimately be reduced to vanishing point. The operating theatre is now regarded as one of the best in Yorkshire, the old theatre having been converted into a children's ward to mitigate the overcrowding there which had made itself apparent. The laundry has been overhauled, and a nursing home with separate bedrooms has been built.

